# Bis[2-hydr­oxy-*N*′-(2-hydroxy­benzo­yl)benzohydrazitato]dipyridine­cadmium(II)

**DOI:** 10.1107/S1600536808034533

**Published:** 2008-10-31

**Authors:** Yu-Ting Chen, Da-Cheng Li

**Affiliations:** a Department of Chemistry, Dezhou University, Dezhou 253023, People’s Republic of China; b College of Chemistry and Chemical Engineering, Liaocheng University, Liaocheng 252059, People’s Republic of China

## Abstract

The title complex, [Cd(C_14_H_11_N_2_O_4_)_2_(C_5_H_5_N)_2_], exhibits crystallographic twofold symmetry. The Cd^II^ atom is located on the twofold rotation axis and reveals a slightly distorted octa­hedral coordination defined by four atoms (N_2_O_2_) from two symmetry-related chelate ligands and two pyridine N atoms. Intra­molecular O—H⋯O and N—H⋯O hydrogen bonds stabilize the mol­ecular conformation while inter­molecular O—H⋯O hydrogen bonding links mol­ecules into a triad, generating a helix along the threefold screw axis.

## Related literature

Three manganese metallacrowns with unsymmetrical aroylhydrazine ligands were synthesized and reported by Dou *et al.* (2006 ▶) and John *et al.* (2006 ▶). For the crystal structure of an iron compound with *N*,*N*′-bis-picolinoyl hydrazine, see: Bernhardt *et al.* (2005 ▶). For a nickel complex formed by *N*,*N*′-disalicyloylhydrazine, see: Chen *et al.* (2007 ▶).
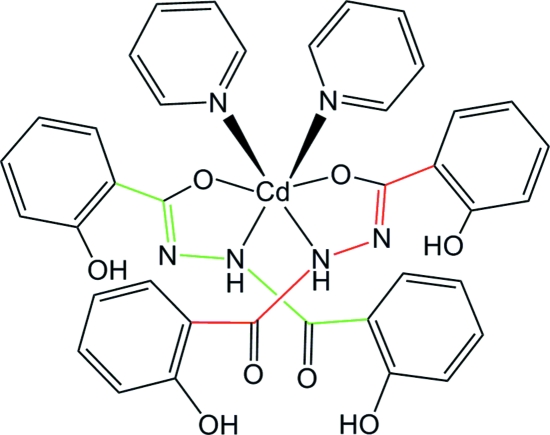


## Experimental

### 

#### Crystal data

[Cd(C_14_H_11_N_2_O_4_)_2_(C_5_H_5_N)_2_]
*M* *_r_* = 813.10
Trigonal, 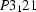

*a* = 13.0380 (10) Å
*c* = 18.069 (3) Å
*V* = 2660.0 (5) Å^3^
*Z* = 3
Mo *K*α radiation
μ = 0.68 mm^−1^
*T* = 298 (2) K
0.40 × 0.38 × 0.35 mm

#### Data collection

Bruker SMART 1000 CCD area-detector diffractometer
Absorption correction: multi-scan (*SADABS*; Sheldrick, 1996 ▶) *T*_min_ = 0.773, *T*_max_ = 0.797
13955 measured reflections
3146 independent reflections
2750 reflections with *I* > 2σ(*I*)
*R*_int_ = 0.033

#### Refinement

*R*[*F* ^2^ > 2σ(*F* ^2^)] = 0.027
*wR*(*F* ^2^) = 0.071
*S* = 1.00
3146 reflections
241 parameters
H-atom parameters constrained
Δρ_max_ = 0.90 e Å^−3^
Δρ_min_ = −0.32 e Å^−3^
Absolute structure: Flack (1983 ▶), 1353 Friedel pairs
Flack parameter: −0.06 (3)

### 

Data collection: *SMART* (Siemens, 1996 ▶); cell refinement: *SAINT* (Siemens, 1996 ▶); data reduction: *SAINT*; program(s) used to solve structure: *SHELXS97* (Sheldrick, 2008 ▶); program(s) used to refine structure: *SHELXL97* (Sheldrick, 2008 ▶); molecular graphics: *SHELXTL* (Sheldrick, 2008 ▶); software used to prepare material for publication: *SHELXTL*.

## Supplementary Material

Crystal structure: contains datablocks I, global. DOI: 10.1107/S1600536808034533/kp2188sup1.cif

Structure factors: contains datablocks I. DOI: 10.1107/S1600536808034533/kp2188Isup2.hkl

Additional supplementary materials:  crystallographic information; 3D view; checkCIF report

## Figures and Tables

**Table 1:** Selected bond lengths (Å)

Cd1—N1	2.331 (3)
Cd1—N3	2.337 (3)
Cd1—O1	2.389 (2)

**Table 2:** Hydrogen-bond geometry (Å, °)

*D*—H⋯*A*	*D*—H	H⋯*A*	*D*⋯*A*	*D*—H⋯*A*
O4—H4⋯O3	0.82	1.92	2.638 (4)	145
N2—H2⋯O2	0.86	1.94	2.624 (4)	135
O2—H2*A*⋯O3^i^	0.82	1.88	2.639 (3)	153

Symmetry code: (i) 
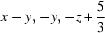
.

**Fig. 1.**
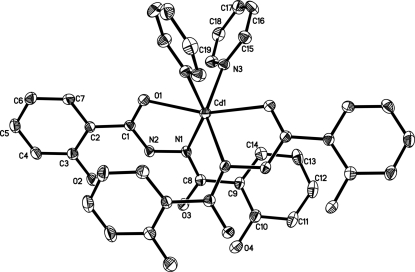
The molecular structure of the title complex. Displacement ellipsoids are drawn at the 30% probability level.

**Fig. 2.**
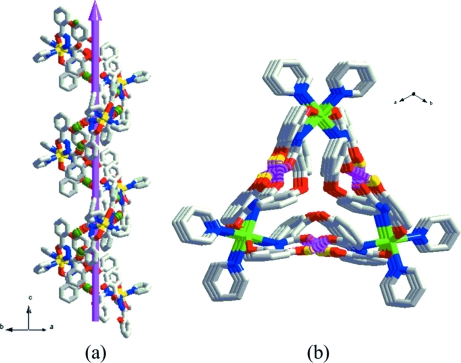
Supramolecular structure of the title complex in the direction [001].

## supplementary crystallographic information

### Comment

Metal complexes with aroylhydrazine ligands are of increasing attention due to their interesting chemical activities (John *et al.* 2006; Dou *et al.*, 2006). However, the research on the compounds with symmetrical diaroylhydrazine ligands was limited (Bernhardt *et al.*, 2005; Chen *et al.*, 2007). As an extension of our work on the structural characterization of these compounds, the title complex, (I), is synthesized and characterized by X-ray structure analysis. The complex (I) exhibits a twofold rotation symmetry. It comprises of one Cd^II^ atom at special position at the twofold rotation axes coordinated by two  ligands and two pyridines (Fig. 1 and Table 1). Each ligand acts as the bidentate via the iminoacyl groups  forming  two five-membered rings around metal ion with the  dihedral angle of 59.71 (4)°.

Intramolecular O4—H4···O3 and N2—H2···O2 hydrogen bonds stabilizes the molecular conformation. There is also an intermolecular hydrogen bond O—H···O hydrogen bond [ 2.639 (3) Å] (Table 2) assembling  three molecules into a triad, that is a basic structural element of a helix along [0 0 1] direction (Fig. 2).

### Experimental

The solution of Cd(NO_3_)_2_4H_2_O (0.123 g, 0.4 mmol) in methanol (10 mL) was added to the mixture of 1,2-disalicyloylhydrazine (0.054 g, 0.2 mmol) and sodium hydroxide (0.032 g, 0.8 mmol) in pyridine (10 mL). A colourless solution was generated after stirring  for two hours at room temperature. The solution was allowed to stand for 2 weeks, whereupon white block crystals were obtained. Yield: 0.058 g, 77%. m. p.> 573 K. Anal. for C_38_H_32_CdN_6_O_8_: Calc. C, 56.08; H, 3.93; N, 10.33; Found: C, 56.54; H, 3.71; N, 10.54%. The No. of CCDC: 686345.

### Refinement

All H atoms were placed in geometrically idealized positions and treated as riding on their parent atoms with C(*sp*_2_ hybrid)-H distances of 0.93Å (*U*_iso_(H)=1.2*U*_eq_(C)).

### Crystal data

**Table:**

[Cd(C_14_H_11_N_2_O_4_)_2_(C_5_H_5_N)_2_]	*D*_x_ = 1.523 Mg m^−^^3^
*M**_r_* = 813.10	Mo *K*α radiation, λ = 0.71073 Å
Trigonal, *P*3_1_21	Cell parameters from 5141 reflections
*a* = 13.038 (1) Å	θ = 2.9–22.9°
*c* = 18.069 (3) Å	µ = 0.68 mm^−^^1^
*V* = 2660.0 (5) Å^3^	*T* = 298 K
*Z* = 3	Block, colourless
*F*(000) = 1242	0.40 × 0.38 × 0.35 mm

### Data collection

**Table:**

Bruker SMART 1000 CCD area-detector diffractometer	3146 independent reflections
Radiation source: fine-focus sealed tube	2750 reflections with *I* > 2σ(*I*)
graphite	*R*_int_ = 0.033
φ and ω scans	θ_max_ = 25.0°, θ_min_ = 1.8°
Absorption correction: multi-scan (SADABS; Sheldrick, 1996)	*h* = −15→15
*T*_min_ = 0.773, *T*_max_ = 0.797	*k* = −15→15
13955 measured reflections	*l* = −21→10

### Refinement

**Table:**

Refinement on *F*^2^	Secondary atom site location: difference Fourier map
Least-squares matrix: full	Hydrogen site location: inferred from neighbouring sites
*R*[*F*^2^ > 2σ(*F*^2^)] = 0.027	H-atom parameters constrained
*wR*(*F*^2^) = 0.071	*w* = 1/[σ^2^(*F*_o_^2^) + (0.041*P*)^2^ + 0.5675*P*] where *P* = (*F*_o_^2^ + 2*F*_c_^2^)/3
*S* = 1.00	(Δ/σ)_max_ < 0.001
3146 reflections	Δρ_max_ = 0.90 e Å^−^^3^
241 parameters	Δρ_min_ = −0.32 e Å^−^^3^
0 restraints	Absolute structure: Flack (1983), 1353 Friedel pairs
Primary atom site location: structure-invariant direct methods	Flack parameter: −0.06 (3)

### Special details

**Table:**

Geometry. All e.s.d.'s (except the e.s.d. in the dihedral angle between two l.s. planes) are estimated using the full covariance matrix. The cell e.s.d.'s are taken into account individually in the estimation of e.s.d.'s in distances, angles and torsion angles; correlations between e.s.d.'s in cell parameters are only used when they are defined by crystal symmetry. An approximate (isotropic) treatment of cell e.s.d.'s is used for estimating e.s.d.'s involving l.s. planes.
Refinement. Refinement of *F*^2^ against ALL reflections. The weighted *R*-factor *wR* and goodness of fit *S* are based on *F*^2^, conventional *R*-factors *R* are based on *F*, with *F* set to zero for negative *F*^2^. The threshold expression of *F*^2^ > σ(*F*^2^) is used only for calculating *R*-factors(gt) *etc*. and is not relevant to the choice of reflections for refinement. *R*-factors based on *F*^2^ are statistically about twice as large as those based on *F*, and *R*- factors based on ALL data will be even larger.

### Fractional atomic coordinates and isotropic or equivalent isotropic displacement parameters (Å^2^)

**Table:**

	*x*	*y*	*z*	*U*_iso_*/*U*_eq_	
Cd1	1.0000	0.45722 (2)	0.6667	0.04088 (11)	
N1	0.8620 (2)	0.2612 (2)	0.69499 (15)	0.0387 (7)	
N2	0.8356 (3)	0.2467 (3)	0.77051 (14)	0.0406 (6)	
H2	0.8005	0.1771	0.7897	0.049*	
N3	0.8726 (2)	0.5186 (3)	0.61934 (16)	0.0449 (7)	
O1	0.9119 (2)	0.4418 (2)	0.78506 (13)	0.0517 (7)	
O2	0.7567 (3)	0.1137 (2)	0.88928 (14)	0.0584 (7)	
H2A	0.7290	0.0561	0.9170	0.088*	
O3	0.8013 (3)	0.06392 (19)	0.69676 (12)	0.0558 (6)	
O4	0.9060 (3)	0.0079 (3)	0.59233 (16)	0.0797 (10)	
H4	0.8760	0.0022	0.6331	0.120*	
C1	0.8648 (3)	0.3405 (3)	0.81227 (18)	0.0391 (8)	
C2	0.8409 (3)	0.3228 (3)	0.89327 (19)	0.0403 (8)	
C3	0.7918 (3)	0.2136 (4)	0.9296 (2)	0.0454 (9)	
C4	0.7786 (4)	0.2086 (4)	1.0058 (2)	0.0548 (10)	
H4A	0.7467	0.1360	1.0298	0.066*	
C5	0.8122 (4)	0.3102 (4)	1.0464 (2)	0.0614 (11)	
H5	0.8012	0.3053	1.0974	0.074*	
C6	0.8618 (4)	0.4184 (4)	1.0120 (2)	0.0556 (10)	
H6	0.8858	0.4872	1.0393	0.067*	
C7	0.8753 (3)	0.4233 (3)	0.9357 (2)	0.0477 (9)	
H7	0.9085	0.4965	0.9123	0.057*	
C8	0.8396 (3)	0.1621 (3)	0.66318 (18)	0.0402 (8)	
C9	0.8647 (3)	0.1680 (3)	0.58281 (18)	0.0415 (8)	
C10	0.9002 (4)	0.0938 (4)	0.5519 (2)	0.0547 (10)	
C11	0.9321 (4)	0.1039 (4)	0.4769 (2)	0.0687 (13)	
H11	0.9592	0.0562	0.4568	0.082*	
C12	0.9225 (4)	0.1852 (4)	0.4338 (2)	0.0673 (12)	
H12	0.9435	0.1926	0.3841	0.081*	
C13	0.8832 (4)	0.2547 (4)	0.4624 (2)	0.0629 (12)	
H13	0.8754	0.3080	0.4320	0.075*	
C14	0.8542 (3)	0.2472 (4)	0.5364 (2)	0.0516 (9)	
H14	0.8274	0.2958	0.5555	0.062*	
C15	0.9145 (4)	0.6185 (4)	0.5806 (2)	0.0550 (10)	
H15	0.9942	0.6587	0.5675	0.066*	
C16	0.8437 (4)	0.6645 (4)	0.5591 (2)	0.0636 (12)	
H16	0.8760	0.7358	0.5334	0.076*	
C17	0.7270 (4)	0.6045 (4)	0.5759 (2)	0.0627 (12)	
H17	0.6777	0.6335	0.5616	0.075*	
C18	0.6827 (3)	0.5002 (4)	0.6144 (2)	0.0591 (11)	
H18	0.6026	0.4570	0.6263	0.071*	
C19	0.7572 (3)	0.4610 (3)	0.6349 (2)	0.0535 (9)	
H19	0.7262	0.3903	0.6612	0.064*	

### Atomic displacement parameters (Å^2^)

**Table:**

	*U*^11^	*U*^22^	*U*^33^	*U*^12^	*U*^13^	*U*^23^
Cd1	0.0445 (2)	0.03796 (14)	0.04239 (18)	0.02224 (11)	0.00869 (17)	0.00434 (9)
N1	0.0392 (17)	0.0368 (16)	0.0340 (15)	0.0144 (14)	0.0019 (12)	0.0033 (12)
N2	0.0437 (16)	0.0396 (16)	0.0347 (15)	0.0179 (13)	0.0072 (13)	0.0058 (14)
N3	0.0448 (18)	0.0430 (17)	0.0494 (17)	0.0236 (15)	0.0064 (13)	0.0045 (14)
O1	0.0692 (18)	0.0398 (14)	0.0459 (15)	0.0271 (13)	0.0147 (13)	0.0067 (12)
O2	0.080 (2)	0.0438 (15)	0.0434 (15)	0.0254 (14)	0.0077 (13)	0.0107 (13)
O3	0.0734 (19)	0.0376 (13)	0.0493 (14)	0.0223 (15)	0.0222 (15)	0.0080 (11)
O4	0.118 (3)	0.081 (2)	0.066 (2)	0.070 (2)	0.0278 (19)	0.0082 (17)
C1	0.0388 (19)	0.041 (2)	0.041 (2)	0.0226 (17)	0.0035 (16)	0.0036 (17)
C2	0.042 (2)	0.049 (2)	0.0364 (18)	0.0282 (17)	0.0029 (15)	0.0027 (16)
C3	0.044 (2)	0.056 (2)	0.042 (2)	0.0290 (18)	0.0009 (16)	0.0031 (18)
C4	0.065 (3)	0.066 (3)	0.042 (2)	0.040 (2)	0.0030 (19)	0.012 (2)
C5	0.074 (3)	0.092 (4)	0.037 (2)	0.055 (3)	0.005 (2)	0.006 (2)
C6	0.064 (3)	0.073 (3)	0.048 (2)	0.048 (2)	−0.0070 (19)	−0.011 (2)
C7	0.055 (2)	0.049 (2)	0.049 (2)	0.0344 (19)	0.0047 (17)	0.0020 (17)
C8	0.0341 (18)	0.0382 (17)	0.0420 (17)	0.0133 (15)	0.0033 (15)	0.0030 (14)
C9	0.038 (2)	0.0391 (17)	0.0363 (16)	0.0113 (17)	0.0027 (16)	−0.0017 (13)
C10	0.063 (3)	0.049 (2)	0.047 (2)	0.025 (2)	0.005 (2)	−0.0025 (18)
C11	0.077 (3)	0.076 (3)	0.046 (2)	0.033 (3)	0.011 (2)	−0.011 (2)
C12	0.065 (3)	0.073 (3)	0.039 (2)	0.016 (2)	0.004 (2)	−0.006 (2)
C13	0.065 (3)	0.061 (2)	0.040 (2)	0.014 (2)	−0.010 (2)	0.0026 (18)
C14	0.047 (2)	0.044 (2)	0.050 (2)	0.0129 (17)	−0.0035 (18)	−0.0011 (19)
C15	0.050 (2)	0.056 (2)	0.060 (3)	0.027 (2)	0.0090 (18)	0.012 (2)
C16	0.069 (3)	0.067 (3)	0.062 (2)	0.040 (2)	0.007 (2)	0.025 (2)
C17	0.065 (3)	0.088 (3)	0.053 (2)	0.051 (3)	−0.006 (2)	0.002 (2)
C18	0.041 (2)	0.074 (3)	0.062 (3)	0.028 (2)	−0.0012 (19)	−0.007 (2)
C19	0.048 (2)	0.044 (2)	0.063 (2)	0.0198 (19)	0.0099 (19)	0.0037 (19)

### Geometric parameters (Å, °)

**Table:**

Cd1—N1	2.331 (3)	C5—H5	0.9300
Cd1—N1^i^	2.331 (3)	C6—C7	1.387 (5)
Cd1—N3^i^	2.337 (3)	C6—H6	0.9300
Cd1—N3	2.337 (3)	C7—H7	0.9300
Cd1—O1	2.389 (2)	C8—C9	1.482 (4)
Cd1—O1^i^	2.389 (2)	C9—C10	1.382 (5)
N1—C8	1.307 (4)	C9—C14	1.389 (5)
N1—N2	1.397 (4)	C10—C11	1.404 (5)
N2—C1	1.320 (4)	C11—C12	1.371 (6)
N2—H2	0.8600	C11—H11	0.9300
N3—C15	1.332 (5)	C12—C13	1.346 (6)
N3—C19	1.333 (5)	C12—H12	0.9300
O1—C1	1.245 (4)	C13—C14	1.380 (5)
O2—C3	1.357 (4)	C13—H13	0.9300
O2—H2A	0.8200	C14—H14	0.9300
O3—C8	1.271 (4)	C15—C16	1.385 (6)
O4—C10	1.371 (5)	C15—H15	0.9300
O4—H4	0.8200	C16—C17	1.352 (6)
C1—C2	1.490 (5)	C16—H16	0.9300
C2—C7	1.385 (5)	C17—C18	1.372 (6)
C2—C3	1.399 (5)	C17—H17	0.9300
C3—C4	1.384 (5)	C18—C19	1.356 (6)
C4—C5	1.380 (6)	C18—H18	0.9300
C4—H4A	0.9300	C19—H19	0.9300
C5—C6	1.372 (6)		
			
N1—Cd1—N1^i^	89.45 (14)	C5—C6—C7	118.8 (4)
N1—Cd1—N3^i^	145.80 (9)	C5—C6—H6	120.6
N1^i^—Cd1—N3^i^	99.48 (10)	C7—C6—H6	120.6
N1—Cd1—N3	99.48 (10)	C2—C7—C6	122.2 (4)
N1^i^—Cd1—N3	145.80 (9)	C2—C7—H7	118.9
N3^i^—Cd1—N3	91.48 (14)	C6—C7—H7	118.9
N1—Cd1—O1	68.64 (9)	O3—C8—N1	124.5 (3)
N1^i^—Cd1—O1	125.88 (9)	O3—C8—C9	119.0 (3)
N3^i^—Cd1—O1	79.62 (9)	N1—C8—C9	116.4 (3)
N3—Cd1—O1	87.84 (9)	C10—C9—C14	117.9 (3)
N1—Cd1—O1^i^	125.88 (9)	C10—C9—C8	120.1 (3)
N1^i^—Cd1—O1^i^	68.64 (9)	C14—C9—C8	121.9 (3)
N3^i^—Cd1—O1^i^	87.84 (9)	O4—C10—C9	122.1 (3)
N3—Cd1—O1^i^	79.62 (9)	O4—C10—C11	117.1 (4)
O1—Cd1—O1^i^	162.04 (12)	C9—C10—C11	120.7 (4)
C8—N1—N2	112.2 (3)	C12—C11—C10	118.9 (4)
C8—N1—Cd1	131.0 (2)	C12—C11—H11	120.5
N2—N1—Cd1	111.44 (19)	C10—C11—H11	120.5
C1—N2—N1	119.7 (3)	C13—C12—C11	121.0 (4)
C1—N2—H2	120.1	C13—C12—H12	119.5
N1—N2—H2	120.1	C11—C12—H12	119.5
C15—N3—C19	117.2 (3)	C12—C13—C14	120.4 (4)
C15—N3—Cd1	120.9 (2)	C12—C13—H13	119.8
C19—N3—Cd1	121.7 (2)	C14—C13—H13	119.8
C1—O1—Cd1	113.9 (2)	C13—C14—C9	120.9 (4)
C3—O2—H2A	109.5	C13—C14—H14	119.6
C10—O4—H4	109.5	C9—C14—H14	119.6
O1—C1—N2	121.1 (3)	N3—C15—C16	122.3 (4)
O1—C1—C2	120.4 (3)	N3—C15—H15	118.8
N2—C1—C2	118.5 (3)	C16—C15—H15	118.8
C7—C2—C3	118.0 (3)	C17—C16—C15	119.2 (4)
C7—C2—C1	117.0 (3)	C17—C16—H16	120.4
C3—C2—C1	124.9 (3)	C15—C16—H16	120.4
O2—C3—C4	121.0 (4)	C16—C17—C18	118.8 (4)
O2—C3—C2	119.2 (3)	C16—C17—H17	120.6
C4—C3—C2	119.8 (4)	C18—C17—H17	120.6
C5—C4—C3	120.8 (4)	C19—C18—C17	119.1 (4)
C5—C4—H4A	119.6	C19—C18—H18	120.5
C3—C4—H4A	119.6	C17—C18—H18	120.5
C6—C5—C4	120.4 (4)	N3—C19—C18	123.3 (4)
C6—C5—H5	119.8	N3—C19—H19	118.3
C4—C5—H5	119.8	C18—C19—H19	118.3
			
N1^i^—Cd1—N1—C8	−39.3 (3)	C7—C2—C3—C4	0.2 (5)
N3^i^—Cd1—N1—C8	−145.5 (3)	C1—C2—C3—C4	177.0 (4)
N3—Cd1—N1—C8	107.5 (3)	O2—C3—C4—C5	−178.7 (4)
O1—Cd1—N1—C8	−168.6 (3)	C2—C3—C4—C5	0.7 (6)
O1^i^—Cd1—N1—C8	23.6 (3)	C3—C4—C5—C6	−1.4 (6)
N1^i^—Cd1—N1—N2	112.3 (2)	C4—C5—C6—C7	1.2 (6)
N3^i^—Cd1—N1—N2	6.1 (3)	C3—C2—C7—C6	−0.4 (5)
N3—Cd1—N1—N2	−100.9 (2)	C1—C2—C7—C6	−177.5 (3)
O1—Cd1—N1—N2	−17.02 (19)	C5—C6—C7—C2	−0.3 (6)
O1^i^—Cd1—N1—N2	175.09 (18)	N2—N1—C8—O3	−1.8 (5)
C8—N1—N2—C1	172.8 (3)	Cd1—N1—C8—O3	149.6 (3)
Cd1—N1—N2—C1	15.7 (3)	N2—N1—C8—C9	180.0 (3)
N1—Cd1—N3—C15	−164.9 (3)	Cd1—N1—C8—C9	−28.6 (4)
N1^i^—Cd1—N3—C15	−61.7 (4)	O3—C8—C9—C10	−30.0 (5)
N3^i^—Cd1—N3—C15	47.6 (3)	N1—C8—C9—C10	148.3 (3)
O1—Cd1—N3—C15	127.1 (3)	O3—C8—C9—C14	150.3 (4)
O1^i^—Cd1—N3—C15	−39.9 (3)	N1—C8—C9—C14	−31.4 (5)
N1—Cd1—N3—C19	20.6 (3)	C14—C9—C10—O4	−175.9 (4)
N1^i^—Cd1—N3—C19	123.8 (3)	C8—C9—C10—O4	4.4 (6)
N3^i^—Cd1—N3—C19	−126.9 (3)	C14—C9—C10—C11	4.1 (6)
O1—Cd1—N3—C19	−47.3 (3)	C8—C9—C10—C11	−175.6 (4)
O1^i^—Cd1—N3—C19	145.6 (3)	O4—C10—C11—C12	177.2 (4)
N1—Cd1—O1—C1	19.5 (2)	C9—C10—C11—C12	−2.9 (7)
N1^i^—Cd1—O1—C1	−53.3 (3)	C10—C11—C12—C13	−0.1 (7)
N3^i^—Cd1—O1—C1	−147.5 (3)	C11—C12—C13—C14	1.6 (7)
N3—Cd1—O1—C1	120.5 (2)	C12—C13—C14—C9	−0.2 (6)
O1^i^—Cd1—O1—C1	166.0 (2)	C10—C9—C14—C13	−2.6 (5)
Cd1—O1—C1—N2	−19.3 (4)	C8—C9—C14—C13	177.1 (3)
Cd1—O1—C1—C2	160.3 (2)	C19—N3—C15—C16	2.3 (6)
N1—N2—C1—O1	2.6 (5)	Cd1—N3—C15—C16	−172.4 (3)
N1—N2—C1—C2	−177.0 (3)	N3—C15—C16—C17	−2.1 (6)
O1—C1—C2—C7	−1.1 (5)	C15—C16—C17—C18	0.6 (6)
N2—C1—C2—C7	178.5 (3)	C16—C17—C18—C19	0.5 (6)
O1—C1—C2—C3	−178.0 (3)	C15—N3—C19—C18	−1.2 (6)
N2—C1—C2—C3	1.6 (5)	Cd1—N3—C19—C18	173.5 (3)
C7—C2—C3—O2	179.6 (3)	C17—C18—C19—N3	−0.2 (7)
C1—C2—C3—O2	−3.5 (5)		

Symmetry codes: (i) −*x*+2, −*x*+*y*+1, −*z*+4/3.

### Hydrogen-bond geometry (Å, °)

**Table:**

*D*—H···*A*	*D*—H	H···*A*	*D*···*A*	*D*—H···*A*
O4—H4···O3	0.82	1.92	2.638 (4)	145
N2—H2···O2	0.86	1.94	2.624 (4)	135
O2—H2A···O3^ii^	0.82	1.88	2.639 (3)	153

Symmetry codes:  (ii) *x*−*y*, −*y*, −*z*+5/3.
